# What are important ingredients for Intensive Home Support for people with severe mental illness according to experts? A concept mapping approach

**DOI:** 10.1186/s12888-023-04975-7

**Published:** 2023-06-28

**Authors:** Caroline van Genk, Diana Roeg, Maaike van Vugt, Jaap van Weeghel, Tine Van Regenmortel

**Affiliations:** 1 Tranzo Scientific Center for Care and Wellbeing, School of Social & Behavioral Sciences, Tilburg University, Tilburg, The Netherlands; 2 Trimbos Institute, Dutch Institute of Mental Health and Addiction, Utrecht, The Netherlands; 3 Faculty of Social Sciences – HIVA, University of Leuven, Leuven, Belgium; 4 Kwintes Housing and Rehabilitation Services, Zeist, The Netherlands; 5 HVO-Querido, Amsterdam, The Netherlands

**Keywords:** Concept mapping, Severe mental illness, Community mental health care, Floating outreach, Intensive home support, Supported housing

## Abstract

**Background:**

Deinstitutionalization in mental health care has been an ongoing process for decades. More and more people with severe mental illness (SMI), who previously lived in residential supported housing settings and were formerly homeless, are now living independently in the community but need intensive support to enable independent living. The support provided by regular outpatient teams is inadequate for this target group. This study explored the ingredients for an alternative form of outpatient support: intensive home support (IHS).

**Methods:**

Concept mapping was used, following five steps: (1) brainstorming, (2) sorting, (3) rating, (4) statistical analysis & visual representation, and (5) interpretation. Purposive sampling was used to represent several perspectives, including researchers, professionals, peer workers, and policy makers.

**Results:**

Experts (n = 17) participated in the brainstorming step and the sorting and rating steps (n = 14). The 84 generated statements were grouped into 10 clusters:. (1) housing rights; (2) informal collaboration; (3) reciprocity in the community; (4) normalization and citizenship; (5) recovery; (6) sustainable funding; (7) equivalence; (8) flexible, proactive 24/7 support; (9) public health and positive health; and (10) integrated cooperation in support at home.

**Conclusions:**

Given the diversity of the ingredients contained in the clusters, it seems that IHS should be designed according to a holistic approach in collaboration with several sectors. Additionally, IHS is not only the responsibility of care organizations but also the responsibility of national and local governments. Further research about collaboration and integrated care is needed to determine how to implement all of the ingredients in practice.

## Background

In many European countries, deinstitutionalization in mental health care has been an ongoing process over the last few decades. This has involved a move away from large psychiatric hospitals to supported accommodation in the 1980s to more independent living in their own homes in the community in the last decades [1]. In recent years, many service providers in Europe have shifted towards meeting individual needs and personal goals from the user’s perspective. The emphasis is now on giving clients more autonomy and decision-making powers [2].Since the 1980s, supported housing (SH) has offered an alternative to long-term residence in a psychiatric hospital [3]. SH aimed to address service users’ functional impairments by helping them to develop practical living skills, improve social functioning, support their way to successful community living, and promote recovery and independence [4, 5]. Originally, there were two forms of supported housing internationally: aggregated settings and floating outreach [6]. In recent years, the aggregated settings have become smaller in scale andwith a greater focus on recovery. Additionally, more and more clients have successfully moved on from residential care or supported housing to more independent accommodation [7]. More intermediate housing forms have also now emerged, including congregate settings, group homes, and satellite homes [8][9–12]There is currently a further development for outflowing residential supported housing clients and formerly homeless people. This group needs more intensive support to enable independent living than a regular floating outreach group, due to their increased psychological and social vulnerability.This results in a higher risk for experienced loneliness and anxiety by the clients, less participation in social activities, and greater feelings of social isolation as a result of living alone [13–17]. In addition, due to long stays in SH settings, this group has become dependent on this environment, which leads to hospitalization and has resulted in reduced self-reliance and independence [18]. The formerly homeless population also needs support in addition to suitable housing to prevent relapse into homelessness and to improve mental health and psychosocial outcomes [19]. For that reason, a new offering of SH was developed to the existing options: intensive home support (IHS) [20]. This support includes the intensive support of a 24/7 supported housing setting but with all of the advantages of living independently in the community and considering the other needs of these clients. Little is yet known in the international literature supported housing [12, 21] and in specific about IHS. Only Independent Supported Housing in Switzerland has many similarities, but also some differences, such as a psychiatrist in the support team to consult if needed [22].

Based on the spare literature about regular floating outreach support and independent living for people with SMI, we can learn the following. Previous research has shown that living independently has many advantages over accommodation based of SH. Floating outreach services provide the greatest opportunity for people with SMI to choose and control their lives [6], which is an important factor in recovery [23]. Furthermore, the cost of floating outreach is much lower than the cost of residential care [6]. Another advantage is having their own home instead of a group housing setting. This is important, as we know that experiencing one’s place of residence as one’s home is associated with more autonomy, empowerment, and personal recovery [6, 24, 25].People also do not have to move if their care needs change as floating outreach is flexible in type and intensity of services [26]. Finally, floating outreach services contribute to reducing hospitalization and increasing housing stability [27]. Previous research has shown that the majority (84%) of service users with SMI also prefer a more independent living modality [13]. In the Netherlands, clients in 75% of shelter and supported housing organizations already receive floating outreach support [28]. Furthermore,Housing First projects and their clients are increasing in numbers [29]. IHS targets individuals moving out from accommodation based supported housing for whom regular outpatient support is not sufficient, new clients that are indicated for IHS, and formerly homeless people with multiple problems.

For all people with SMI who desire to live independently or already live independently with too little floating outreach support, IHS complements existing offerings, in addition to the multidisciplinary Flexible Assertive Community Treatment (FACT) teams from the mental health care sector. Both have a focus on symptomatic, personal, and societal recovery [4, 30], but FACT from a treatment perspective and IHS from a (housing) support perspective. IHS and FACT are also funded in different ways [20]. No previous research has yet examined how this support should be designed to serve the clients in the best way. This paper, therefore, considers what the essential ingredients and preconditions for IHS should be. The key research question of this study is: what are the essential ingredients of IHS according to experts?

## Methods

### Concept Mapping

Our main interest was exploring the ingredients of IHS for people with SMI by Dutch experts in the field. To achieve this, we used concept mapping as a research method as described by Kane and Trochim [31]. This approach included five steps: (1) brainstorming, (2) rating, (3) sorting, (4) statistical analysis & visual representation, and (5) interpretation. The analysis process consisted of quantitative techniques of multi-dimensional scaling and hierarchical cluster analyses and helped interpret the data by producing visual maps [31]. We used the software program Group Wisdom for steps 2, 3, and 4 of the concept mapping [32]. All statements and cluster names were translated into English for this paper by a professional translator.

### Participants

The participants were selected through purposive sampling [33]. This ensured the inclusion of many different viewpoints. We aimed for a wide and diverse group, with a size of between 10 and 20 participants [34]. The research team made a list of relevant experts, providing a good overview of the national experts in this area. The participants follow developments in the field or implement this development in practice. They have a view of IHS in various ways: scientific, political, educational, managerial, and practical. Most participants have years of experience in treatment, de-institutionalization, persons with SMI, and expertise in the field of IHS. For example, several participants have/had a supporting role in developing and implementing IHS, after years of expertise in supported housing as a professional in several locations. Some have moved to an advisory role within the supported housing. Additionally, two board members from supported housing organizations participated. Three peer supporters also participated, including one peer supporter in homelessness and shelter. One person performed her PhD on supported housing and recovery, as well as trained as a health care professional and now works as a policy maker in a mental health care institute. Several experts are known as progressive in the field of client-centered mental health care innovations, for example as advisors to the government and founder of a recovery college. The professors who participated have years of experience in research, such as on deinstitutionalization and innovation in mental health care. Finally, several psychologists and psychiatrists participated who work with the target group daily. With this, there is a lot of recovery expertise in the group of participants. The seventeen participants gave their informed consent.

**Table 1:** Participant Demographic Characteristics

		Participants brainstorming session (*n* = 17)	Participants brainstorming, sorting & rating (*n =* 14)
Gender	Male	8	7
	Female	9	7
Function	Board members of supported housing organizations	2	1
	Researchers	3	3
	Policy makers	5	4
	Professionals	4	4
	Different	3	2

### Procedure

#### Brainstorming

The brainstorming step aimed to collect a wide range of statements regarding the subject—in this case, the ingredients of IHS for people with SMI. The session started with a short presentation about the subject. Thereafter, brainstorming was guided by the prompt: “The ingredients of Intensive Home Support are….” During the process of generating statements, every participant could give their contribution and were allowed to suggest as many ideas as possible. Other participants were allowed to respond, but not comment about the relevance of the statements. Every response about the subject was treated as valid. All statements appeared immediately on the screen, and the participants checked whether their statements were noted correctly.

#### Sorting and rating

The participants were asked to sort, individually, the brainstormed statements into groups based on conceptual similarity and to provide a name for each group. The participants then rated all statements in terms of importance, on a Likert scale from 1 (not important) to 5 (very important).

#### Analysis

After the sorting and rating steps, we started the analysis. The analysis process generated a “group product” consisting of visual maps, which were easy to understand and evaluate during the interpretation phase. Group Wisdom also calculated the bridging values (BV) for individual statements and clusters, as well as the mean and standard deviation only for the clusters. The BV refers to how the statements are related to other statements and ranges from 0 to 1. The lower the BV, the more the statement is anchored to its place on the map, meaning that it has been sorted with statements that are in the same area on the map. The higher the BV, the more a statement has been sorted with statements placed further away on the map, thereby bridging it to other areas on the map. Statements with a low BV are more representative of the meaning of the cluster in which they are located than those with higher BVs. The BV for a cluster is the average of all the BVs for the statements in each cluster. Lower BVs represent a more homogeneous cluster and higher BVs a more heterogeneous cluster [31]. We then calculated the stress value, which indicates the goodness of fit of the configuration, with lower stress values having a better fit [31], with a maximum of 0.365 [35].

#### Interpretation

After the analysis, the maps were presented in an online meeting to the participants. This involved a group discussion to stimulate responses and reach consensus about the cluster names. Thereafter, the axes were labeled from top to bottom and from left to right. The axes provide insight into how the clusters relate to each other. Finally, possible explanations for the statements and clusters were discussed.

## Results

**Fig. 1:**
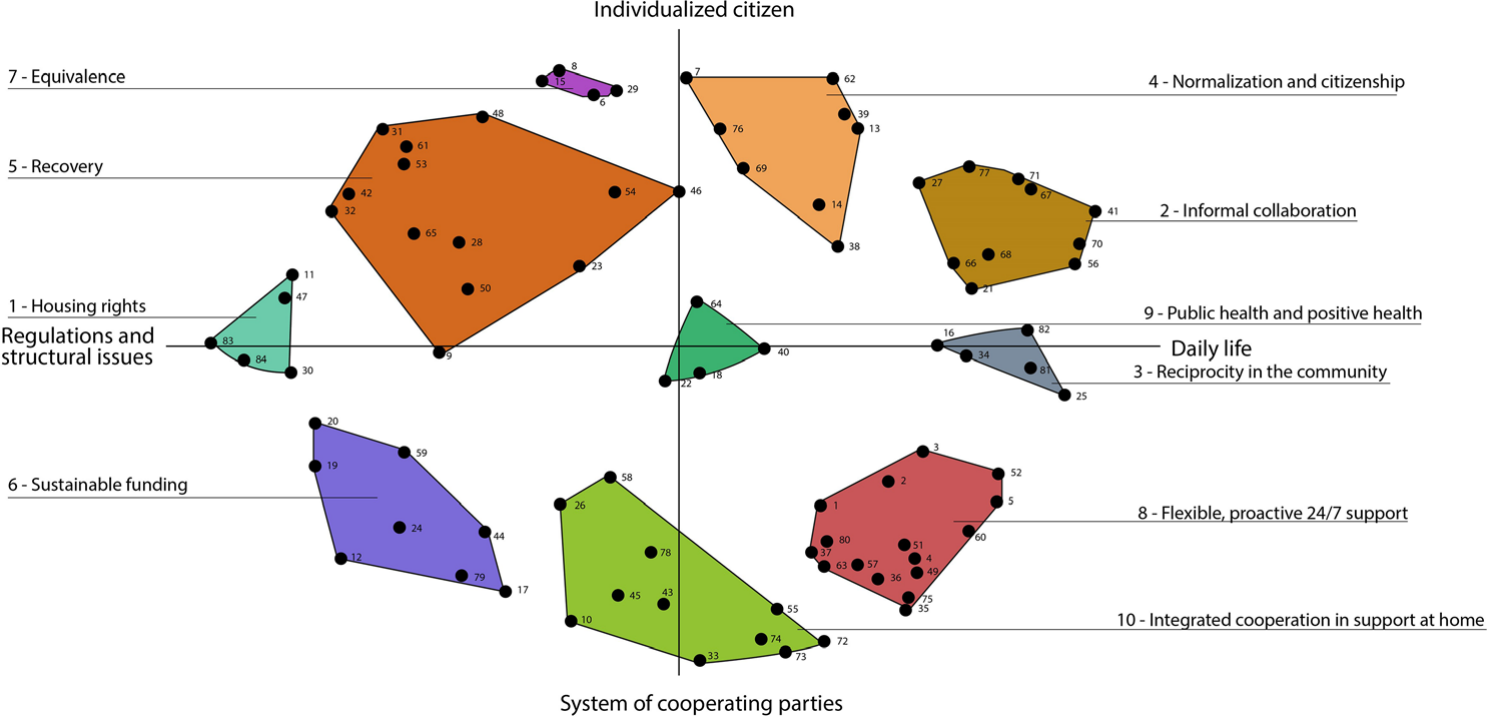
Cluster map

**Fig. 2:**
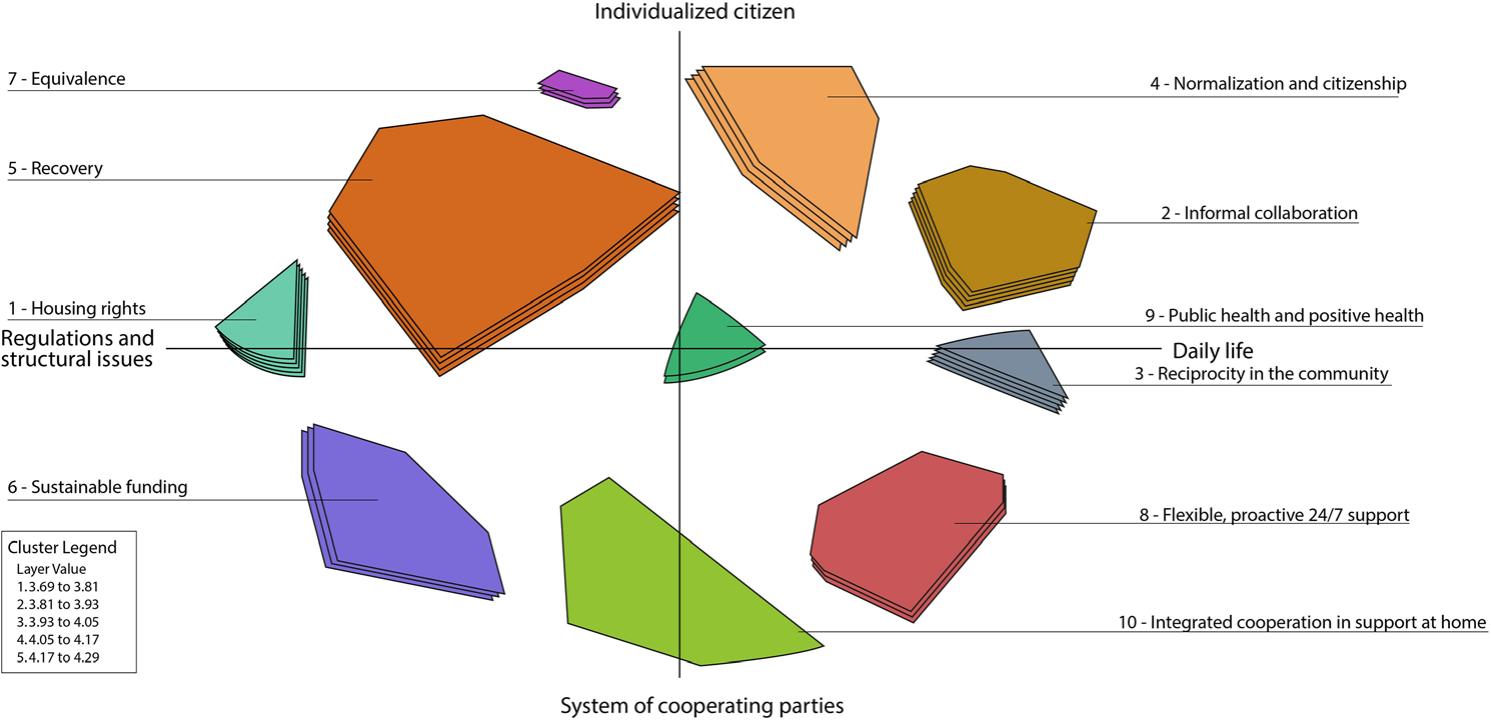
Cluster rating map

A total of 91 statements were generated by 17 participants (Table 1). After removing duplicates, 84 unique statements remained. The 84 statements are divided into 10 clusters, shown in Table 2 with the mean ratings for importance and BVs, with the most prioritized statements in italics. These clusters were based on their individual rated and sorted data. Figure 1 shows the cluster map (the point map with the 10 clusters) and dimensions. Figure 2 shows the cluster rating map. To enhance the understanding of the results of this study, this section includes four parts: (1) importance, (2) bridging values, (3) an overview of clusters, and (4) the signification of the axes in the concept map.

**Table 2:** Statements grouped by clusters with bridging values and importance rating

*Cluster 1 – housing rights*		BV	Rating mean (SD)*
	Importance Rating Values Results		4.17–4.29
Nr.	Bridging Values Cluster	0.78	
83.	*Housing rights*	0.62	4.57 (0.62)
84.	*Your own home*	0.96	4.57 (0.49)
30.	Housing is a fundamental right, even when things go wrong in a home, a client must continue to have the right to a home	0.66	4.50 (0.63)
47.	Review of concept of self-reliance by municipalities due to high expectations of citizens’ self-reliance in general	0.87	3.86 (0.91)
11.	Attention to entering into dialogue about the enormous growth of compulsion and coercion in the home situation	0.77	3.39 (0.80)
***Cluster 2 – informal collaboration***			
	Importance Rating Values Results		4.17–4.29
Nr.	Bridging Values Results	0.40	
41.	*Cooperation with and positioning of relatives*	0.51	4.57 (0.49)
70.	*Involving loved ones*	0.38	4.57 (0.49)
68.	Involving peer support	0.46	4.50 (0.63)
21.	Exchange of people and neighborhood, also positive exchange	0.31	4.36 (0.72)
66.	Protective factors: social network, close relatives, employment, and participation	0.48	4.29 (0.45)
71.	Working with Resource Groups	0.27	4.29 (0.59)
77.	A method in which the person determines who is involved, as in Open Dialogue	0.43	4.29 (0.45)
56.	Going beyond linking with the network, what can the informal network do concretely?	0.36	4.21 (0.56)
67.	Involving family experience experts in Resource Groups	0.29	3.86 (0.83)
27.	Scope for citizen- and consumer-run initiatives	0.50	3.79 (1.21)
***Cluster 3 – reciprocity in the community***			
	Importance Rating Values Results		4.17–4.29
Nr.	Bridging Values Results	0.43	
25.	Presence available when the need arises, also by neighborhood and family	0.43	4.43 (0.49)
82.	Organizing link with the neighborhood	0.44	4.43 (0.49)
81.	Opening existing facilities such as a community center in the neighborhood to everyone	0.48	4.29 (0.59)
16.	How can the mental health care sector, social domain, and network be more reciprocal/serving to the community	0.37	4.07 (0.88)
34.	Healthcare providers must contribute to the neighborhood community and not only request	0.41	4.07 (0.7)
***Cluster 4 – normalization and citizenship***			
	Importance Rating Values Results		4.05–4.17
Nr.	Bridging Values Results	0.49	
69.	*Using recovery-oriented work as a methodology*	0.51	4.57 (0.62)
13.	Open dialogue on autonomy/shared control	0.51	4.36 (0.61)
39.	Investing in discussing how to reach an agreement on recovery goals with different parties	0.54	4.29 (0.45)
76.	Not treatment goals, but the goals of the person concerned are leading	0.55	4.21 (0.56)
38.	An open discussion between all parties about the risks we do/do not want to take	0.45	4.21 (0.41)
14.	Make sure we transcend the patient role and just be neighbors	0.42	4.14 (0.52)
62.	Knowledge of the environment regarding how to deal with people with a vulnerability such as through mental health first aid training	0.54	3.79 (0.41)
7.	We are all a bit crazy, and craziness has an added value too	0.38	3.64 (1.04)
***Cluster 5 - recovery***			
	Importance Rating Values Results		4.05–4.17
Nr.	Bridging Values Results	0.57	
28.	Facilitating people’s autonomy	0.57	4.50 (0.73)
31.	Rights such as participation are basic principles, not favors	0.49	4.50 (0.63)
42.	Designing the curriculum of the Social Work program (senior secondary vocational education (MBO) & universities of applied sciences (HBO)) to focus on recovery and network psychiatry	0.82	4.50 (0.50)
65.	A good vision of citizenship and human rights in governments and organizations, care and welfare organizations, and the mental health care sector	0.49	4.36 (0.81)
61.	Focus on health rather than on illness or disability	0.49	4.29 (0.70)
50.	Attention to individual housing needs, such as the need for or lack of stimuli	0.62	4.29 (0.59)
46.	Normalizing vulnerability because no one is capable of complete self-reliance	0.43	4.14 (0.64)
53.	Positive attention, give people energy to take a new step	0.48	4.14 (0.64)
48.	Good balance between risk and growth of clients by care providers	0.54	4.00 (0.53)
32.	Recovery must also become the guiding principle in nursing education	0.77	3.93 (0.59)
23.	Seeing it as a public resource, normalizing it, and making it available to all	0.41	3.64 (0.89)
54.	Watchful Waiting because a proportion of people with mental health problems recover spontaneously	0.51	3.57 (0.49)
9.	Care providers must give up control to parties in a vulnerable position	0.74	3.14 (0.52)
***Cluster 6 – sustainable funding***			
	Importance Rating Values Results		3.93–4.05
Nr.	Bridging Value Cluster	0.73	
19.	A pleasant place that feels like home	0.94	4.36 (0.61)
59.	Well-qualified staff who also look at the contribution of the team when the client is not doing well	0.84	4.21 (0.56)
12.	Full range instead of a meager product that must not cost too much	0.81	4.14 (0.74)
79.	Decompartmentalization of the financial flows	0.55	4.14 (0.91)
44.	Person-driven funding: does the client benefit, instead of fixed product	0.51	4.07 (0.80)
17.	The mental health care sector must have a more serving role instead of being a claimant in shared principles with the social domain	0.50	3.86 (0.99)
20.	Focus on safety in the home situation	1.00	3.79 (0.56)
24.	Deploying Intensive Home Support based on people’s needs and not because of housing shortages	0.67	3.79 (1.15)
***Cluster 7 - equivalence***			
	Importance Rating Values Results		3.93–4.05
Nr.	Bridging Values Results	0.39	
29.	*Seeing the client not only as a person who needs help but also as a person who has something to offer*	0.30	4.64 (0.48)
15.	It is about ordinary life wishes	0.47	4.07 (0.80)
6.	Recognizing another in being different	0.37	4.00 (1.00)
8.	Normalizing problem behavior by replacing the term autonomy with the term shared control/ownership	0.40	3.36 (1.11)
***Cluster 8 – flexible, proactive 24/7 support***			
	Importance Rating Values Results		3.93–4.05
Nr.	Bridging Values Results	0.21	
51.	Flexibility regarding time and hours and type of support	0.16	4.50 (0.63)
37.	Flexible, continuous, and close support	0.10	4.29 (0.8)
80.	Cooperation between supported housing supervision and any mental health care practitioners	0.11	4.29 (0.88)
5.	People can engage support themselves, including at night and on weekends	0.40	4.21 (0.41)
2.	Offering process guidance, job coaching, and daytime activities	0.33	4.14 (0.74)
63.	Good options in case of a crisis with 24-hour access to someone to talk to and the use of a crisis card	0.02	4.07 (0.46)
4.	Availability of an on-call service	0.07	4.07 (0.59)
52.	Also, pay attention when the client apparently asks for little help	0.51	4.07 (0.59)
57.	Scaling up in case of crisis, if necessary to Intensive Home Treatment level	0.00	4.07 (0.46)
60.	Good 24-hour accessibility of care, not only in the context of a crisis	0.28	4.00 (0.38)
75.	Cooperation with FACT	0.15	4.00 (0.76)
35.	Possibility for the client to seek contact and proximity to the support	0.34	3.93 (0.46)
3.	Also, provide support to people who do not have a request yet but need support	0.37	3.93 (0.46)
1.	Scaling down to outpatient support if someone is sufficiently independent that he/she makes less use of the comprehensive decision on the care required	0.16	3.71 (0.59)
49.	Preparing clients at supported accommodations in time for their desire to live independently	0.17	3.64 (1.04)
36.	Long-term support by a fixed team	0.22	3.43 (0.73)
***Cluster 9 - public health and positive health***			
	Importance Rating Values Results		3.81–3.93
Nr.	Bridging Values Results	0.33	
18.	Lifestyle interventions as a method of strong cooperation between the mental health care sector and social domain aimed at fulfillment	0.34	4.14 (0.64)
64.	Good access to comprehensive healthcare from a positive health vision for all citizens and all aspects of health	0.37	4.14 (0.91)
40.	Rediscovering and modernising Kwartiermaken (quarter-making) (after the book by Doortje Kal)	0.30	3.79 (1.15)
22.	Setting up as public care (in all areas, without selection or indication)	0.32	3.36 (1.11)
***Cluster 10 - integrated cooperation in support at home***			
	Importance Rating Values Results		3.69–3.81
Nr.	Bridging Values Results	0.29	
78.	*Decompartmentalization of different cultures of the mental health care sector, supported housing and social domain to promote cooperation*	0.26	4.57 (0.49)
58.	Do not tie everything down in a product description so that there is room to do what is needed at the moment	0.37	4.00 (0.65)
55.	A large number of hours of support per week is possible with room for scaling up when and for whom needed	0.15	3.93 (0.59)
33.	View Intensive Home Support as a supplement to normal WMO support and not only as an outflow from supported housing	0.35	3.86 (0.99)
73.	Cooperation with housing assistance	0.25	3.79 (0.77)
74.	Cooperation with supported housing	0.26	3.79 (0.77)
26.	Preventing it from becoming compartmentalized but accessible to everyone in society	0.47	3.71 (0.8)
72.	Falling back on a supported housing facility for company or a question	0.19	3.36 (0.81)
10.	The mental health care sector is in a position of power and must give up budget to the community to make self-organization possible	0.41	3.29 (0.96)
43.	Control support by specialized supported housing teams instead of in the mental health care	0.20	3.14 (0.99)
45.	The future of supported housing and Intensive Home Support to be placed in the social domain instead of the mental health care sector	0.26	3.14 (1.06)
*Rating: vary from 1 (not important) to 5 (very important)			

### Importance

On the 5-point scale (ranging from 1 to 5), the mean ratings of statement importance ranged from 3.14 to 4.64, and all clusters had statements rated above 4.00. The top 7 ratings (all over 4.57) were for statements in clusters 10 (integrated cooperation in support at home), 4 (normalization and citizenship), 2 (informal collaboration), 7 (equivalence), and 1 (housing rights) (see Table 2). Many of these statements were related to the right to a home, working with loved ones, recovery, and seeing the client as a human being with something to offer. The clusters that were prioritized highest, on average, were 1 (housing rights), 2 (informal collaboration), and 3 (reciprocity in the community) (see Fig. 2).

### Bridging values

Clusters 8 (flexible, proactive 24/7 support) and 10 (integrated cooperation in support at home) had the lowest BVs (e.g., 0.21 and 0.29). The low BV shows that the statements in these clusters were often grouped by the participants. Clusters 1 (housing rights) and 6 (sustainable funding) had the highest BVs (e.g., 0.78 and 0.73), which indicates that the statements in these clusters were not often grouped by the participants.

### An overview of the clusters

Group Wisdom produced sample cluster maps with 5 to 15 clusters. The core research group selected the 10-cluster map because the software program merged clusters that had nothing to do with each other beginning with the 9-cluster map. We presented the 10-cluster map to the participants during the interpretation meeting; the participants, in consensus, assigned the 10 clusters the following names: (1) housing rights; (2) informal collaboration; (3) reciprocity in the community; (4) normalization and citizenship; (5) recovery; (6) sustainable funding; (7) equivalence; (8) flexible, proactive 24/7 support; (9) public health and positive health; and (10) integrated cooperation in support at home The clusters correlated reasonably well with each other according to the stress value of 0.3092. The clusters are arranged in order from high priority (#1) to lower priority (#10).

#### Cluster 1: housing rights

The first cluster contains five statements with a BV of 0.78. Housing is central to this cluster: having one’s own home and housing is a fundamental right, even when things go wrong. Also, coercion and compulsion in the home situation and self-reliance are themes in the statements. The highest-rated statements were (83) *housing rights* and (84) *your own home.*

#### Cluster 2: Informal collaboration

The second cluster contains 10 statements about involving significant others (in resource groups) and (family) peer support workers. The highest-rated statements were 41 (*cooperation with and positioning of relatives*) and 70 (*involving loved ones*). The BV of this cluster is 0.40.

#### Cluster 3: reciprocity in the community

The five statements in this cluster defined aspects of connection with the community and how care can become more reciprocal to society. Statements 25 (*presence available when the need arises, also by neighborhood and family*) and 82 (*organizing link with the neighborhood*) received the highest importance rating scores within this cluster. This cluster was, on average, given the highest priority. The BV is 0.43.

#### Cluster 4: normalization and citizenship

The fourth cluster contains eight statements and is situated at the top of the map (orange). All kinds of statements are included in this cluster, such as recovery, citizenship, risk-taking, and autonomy. The highest-rated statements were (69) *using recovery-oriented work as a methodology* and [13] *open dialogue on autonomy/shared control.* The BV of this cluster is 0.49.

#### Cluster 5: recovery

The 13 statements in this large cluster defined aspects of health instead of illness, recovery as a guiding principle in nursing and social work courses, and the normalization of vulnerability. This cluster also contains statements about facilitating autonomy and seeing participation as a starting point rather than favoring it. Statements 28 (*facilitating people’s autonomy*) and 31 (*rights such as participation are basic principles, not favors)* received the highest importance rating scores within this cluster. The BV of this cluster is 0.57.

#### Cluster 6: sustainable funding

The content of the statements in this cluster varied from person-driven funding to full range instead of a poor product, decompartmentalizing financial flows, and focusing on safety at home. Statements 19 (*a pleasant place that feels like home*) and 59 (*well-qualified staff who also look at the contribution of the team when the client is not doing well*) received the highest importance rating scores within this cluster. The BV of this cluster is 0.73.

#### Cluster 7: equivalence

The seventh cluster contains statements about recognizing another in being different, ordinary life desires, and seeing the client not only as a person seeking help but also as someone who has something to offer. Statements 29 (*it is about ordinary life wishes*) and 15 (s*eeing the client not only as a person who needs help but also as a person who has something to offer*) received the highest importance rating scores within this cluster. The BV of this cluster is 0.39.

#### Cluster 8: flexible, proactive 24/7 support

The 16 statements in this cluster defined aspects of the organization of support. The statements reflected the flexibility of support that is available 24/7, easily accessible for clients, and what is needed in crises. Some of the statements thus reflected how to collaborate with other organizations and teams. The highest-rated statements were, respectively, (51) *flexibility regarding time and hours and type of support*; [37] *flexible, continuous, and close support*; and (80) *cooperation between supported housing supervision and any mental health care practitioners.* The BV is 0.21.

#### Cluster 9: public health and positive health

This cluster highlights lifestyle interventions, quarter-making that contributes to the social inclusion of vulnerable people in society, positive health, and good access to comprehensive health care. Statements 18 (*lifestyle interventions as a method in strong cooperation between the mental health care sector and social domain aimed at fulfillment*) and 64 (*good access to comprehensive health care from a positive health vision for all citizens and all aspects of health*) received the highest importance rating scores within this cluster. The BV of this cluster is 0.33.

#### Cluster 10: Integrated cooperation in support at home

The statements in this cluster are closely related to the statements in the eight cluster, but the difference is the focus on intersectoral collaboration between the social domain and the mental health care sector in this cluster, as well as their responsibilities and tasks. Another central element in this cluster is the collaboration with supported accommodation services. Statements 58 (*do not tie everything down in a product description so that there is room to do what is needed at the moment*) and 78 *(decompartmentalization of different cultures of the mental health care sector, supported housing and social domain to promote cooperation)* received the highest importance rating scores within this cluster. The BV is 0.29.

### The signification of axes in the concept map

During the interpretation session, the concept map axes were named, providing insight into the dimensions the participants used to sort the statements. The x-axis in this map (Fig. 1) represents a continuum between r*egulations and structural issues* and *daily life*. The y-axis in this map represents a continuum between *individualized citizen* and *system of cooperating parties*. These axes divide the map into four quadrants, with the top left clusters related to the person him/herself (clusters 7, 5, and 1); the top right clusters concern the person in relation to his/her social network (clusters 4, 3, and 2); the bottom right quadrant focuses on care seen from the perspective of the professional (clusters 8 and 10); and finally, the bottom left quadrant is where the system with its preconditions is situated (cluster 6). Cluster 9 (public health and positive health) is situated at the intersection of the axes.

## Discussion

Using the concept mapping method, this paper explored what is needed to realize IHS and what this support should look like in practice. In total, we found 10 clusters. All of the clusters together seem to constitute IHS, with public health as the central focus that should be accessible to all people [36].

Clusters 3 (reciprocity in the community), 2 (informal collaboration), and 1 (housing rights) were prioritized with the highest importance ratings. Cluster 10 (integrated cooperation in support at home) was the cluster with the lowest priority. On the statement level, statements about collaboration with loved ones (41 + 70), recovery (69), and housing rights (83 + 84) were considered most important by the experts and also correspond to the clusters with the highest importance rating. On a scale of 1 to 5, these differences are minimal, so we will not use the importance ratings in assessing which ingredients are most important for IHS. The clusters together form the ingredients for IHS from the experts’ view.

Even so, the ingredients and clusters were found to differ in the level of implementation. Several parties are responsible for the different ingredients. Some ingredients are the responsibility of health care facilities, such as cluster 8 (flexible, proactive 24/7 support) and cluster 10 (integrated cooperation in support at home), but cluster 4 (normalization and citizenship) and cluster 1 (housing rights), the upper left corner of the map, include conditions that should be created by the community, government, and municipalities. This makes it clear that intersectoral coordination and collaboration are needed to realize IHS. This aligns with the recent conceptualization of network psychiatry in the Netherlands. Network psychiatry seeks to provide a solid, flexible collaboration between multiple disciplines, both the mental health care sector and the social domain, that collaborate effectively with the client, their social network, and with each other [37]. Likewise, the position paper by Keet et al. [38] about the principles and key elements of high-quality, community-based mental health care has common ground with the findings in our study. They recommend a community network of care, as formulated by Trainor and Church [39] as a network that operates within a broader network of self-help, family, friends, and other informal resources and generic community services. This shows that a community care network has been recommended for decades.

Other principles and key elements from Keet et al. [38] are, for instance, the appreciation and use of peer support, public health, recovery, and respect for human rights, and these elements also correspond to the clusters in our study. However, peer support does not directly stand out in the cluster names but was mentioned in two statements in cluster 2 about informal collaboration that received high importance ratings. The use of peer support is necessary if one wants to provide support based on recovery, empowerment, and equality [40]. Peer support can also contribute to finding a connection between clients and caregivers. It is often the peer supporter who can best relate to the client’s situation and thereby gain trust [41]. Hence, peer support should also be considered as a key ingredient for IHS.

Additionally, before we started this concept mapping study, we conducted a scoping review to discover the critical ingredients for community-based mental health support in the scientific literature (publication in preparation). Most of the ingredients of the concept map correspond to the critical ingredients we extracted from the literature: recovery, sustainable financing, reciprocity, and citizenship were both found in the literature and as ingredients in the cluster map. However, although the literature suggests that the use of new technology and multidisciplinary teams are important for community mental health support, in our concept mapping study, these ingredients were not mentioned by the experts. For that reason, our scoping review is a good addition to the experts’ perspective.

Further, we also compared our results with a similar concept mapping study. In 2005, a concept map was created by van Weeghel et al. [42] on the components of good community care. After 17 years, it is striking that this concept map still has many similarities with our concept map, such as recovery, the need for informal caregivers, and tailored care focusing on empowerment. Additionally, in our concept map, there seems to be more focus on innovative topics—human rights, housing rights, public health, and citizenship—than in the concept map of van Weeghel et al. [42]. Housing rights are currently receiving a lot of attention from the Dutch government, and it is also the responsibility of the government to ensure that all citizens have access to good housing. After all, even for people who move into independent living after an episode of homelessness, it is true that appropriate support in their living environment contributes best to (social) recovery. It is therefore crucial that the approach to homelessness with the decentralization of supported housing to IHS is viewed in context [43]. Housing is currently a big challenge because of the major housing shortage, especially in social rental housing, on which our target group often depends when they want to leave supported housing settings. This may cause a delay in the deinstitutionalization process [43], but it is also a prerequisite for IHS to succeed.

We have learned from this concept map and the comparison with the literature that several factors will influence the success of IHS. More attention should be paid to intersectoral collaboration between the government, municipalities, care organizations, welfare organizations, health insurance companies, and housing collaboratives. The financial side of IHS also makes collaboration complex, because the (limited) funding currently lies with the various municipalities and not with the national government in the Netherlands. Furthermore, the different dimensions show the interplay on the micro, meso, and macro levels—that is, respectively, between the individual, his/her social network, the care organizations, and society [36]. Also highlighted are structural issues that must be arranged and for which not only the care organization is responsible, but also the (national) government and the whole society, such as housing rights. This illustrates the complexity of IHS. Consequently, in practice, IHS will be a collaborative product between (social) partners and supported housing organizations in the community.

Finally, it is notable that many statements were phrased abstractly. Concrete, care-related ingredients for daily support by social workers are scant in the findings of this study. All of the statements were formulated by experts who have not themselves received or experienced this support. However, all experts have years of experience concerning outpatient support and supported housing. Nevertheless, the perspective of daily practice is underexposed. This is a gap that will be filled by our follow-up study, in which the clients are asked about their experience with IHS and mention concrete elements that have worked for them during their received support. That study will be a good complement to this concept mapping study.Strengths and limitations.

This study has several strengths. In the first place, at the meetings, all participants were present at the same time. As a result, everyone heard the same presentation and each other’s input and had the chance to respond to it at the same time. This ensured uniformity and transparency. In addition, we invited participants from the entire field, which enabled us to obtain data from different perspectives. Much research has been conducted from the mental health care sector perspective, while the social domain is still understudied in this field of research. We, therefore, paid extra attention to inviting participants who represent the social domain. A final strength is the software program Group Wisdom. The program provided the freedom to the participants to conduct the sorting and ranking part in their own time and at their own pace. However, participants had to be given a clear deadline and clear instructions beforehand.

We also found some limitations. Although we did invite several (policy) staff from the municipalities, they did not participate in the meetings. They gave several reasons why they did not want to participate, such as being too busy, having no interest in participating, and not being involved enough in the topic. This was very unfortunate for this research because the municipalities are responsible for the quality assessment and funding of this care in the Netherlands.- Nevertheless, the healthcare organizations design and perform the services, so it can be assumed that the most relevant ideas were captured. Another limitation is the lack of the client’s perspective. We chose to invite only professionals for the concept mapping because the clients’ perspective will be addressed in the next sub-study associated with this broader study. However, two experts who participated are also family experience experts. Finally, Group Wisdom does not make it possible to rank all statements from not important to very important, but rather all statements had to be ranked individually. As a result, most statements have a high average, and it is difficult to compare which statements were considered the most important. Care must therefore be taken when concluding.

## Conclusions

With this study, we have tried to create an overview of the essential ingredients for IHS for people with SMI according to Dutch experts. Given the diversity of the ingredients contained in the clusters, it seems that IHS should be designed according to a holistic approach involving all caregivers around the client. Additionally, IHS is not only the responsibility of the care organizations but also the responsibility of the national and local governments, which highlights the need to shape IHS in collaboration with the different sectors. Further research about collaboration and integrated care is needed to determine how to implement all of the ingredients in practice, as well as to investigate the perspective of the clients who receive this support in our other sub-study of this doctoral research. All studies together can contribute to the development of a fidelity scale for the implementation of IHS in future research. This makes this study the first step toward investigating whether IHS works in practice.

## Data Availability

The data used and analyzed during the current study are available from the corresponding author upon reasonable request.

## Abbreviations

FACT: Flexible Assertive Community Treatment
IHS: Intensive Home Support
SMI: Severe Mental Illness
SH: Supported Housing
